# General and tuberculosis-specific service readiness in two states in Nigeria

**DOI:** 10.1186/s12913-020-05626-3

**Published:** 2020-08-26

**Authors:** Mojisola Morenike Oluwasanu, Abiodun Hassan, Ayodeji Matthew Adebayo, Queen Chidinma Ogbuji, Bamidele Olaiya Adeniyi, David Ayobami Adewole, Oladapo Alabi Ladipo, Grace Ada Ajuwon, Ademola Ajuwon

**Affiliations:** 1 Department of Health Promotion and Education, African Regional Health Education Centre, Faculty of Public Health, College of Medicine, University of Ibadan, Ibadan, Nigeria; 2 Association for Reproductive and Family Health, Abuja, Nigeria; 3 Department of Community Medicine, Faculty of Clinical Sciences, College of Medicine, University of Ibadan, Ibadan, Nigeria; 4 Federal Medical Centre, Owo, Ondo State Nigeria; 5 Department of Health Policy and Management, Faculty of Public Health, College of Medicine, University of Ibadan, Ibadan, Nigeria; 6 E. Latunde Odeku Medical Library, College of Medicine, University of Ibadan, Ibadan, Oyo State, Nigeria

**Keywords:** Service readiness, Health systems, Tuberculosis, Health workers, Human resources for health

## Abstract

**Background:**

Tuberculosis is the world’s deadliest infectious disease and a leading cause of death in Nigeria. The availability of a functional healthcare system is critical for effective TB service delivery and attainment of national and global targets. This study was designed to assess readiness for TB service delivery in Oyo and Anambra states of Nigeria.

**Methods:**

This was a facility-based study with a mixed-methods convergent parallel design. A multi-stage sampling technique was used to select 42 primary, secondary, and tertiary healthcare facilities in two TB high burden states. Data were collected using key informant interviews, a semi-structured instrument adapted from the WHO Service Availability and Readiness Assessment tool and facility observation using a checklist. Quantitative data were analysed using descriptive and inferential statistics while qualitative data were transcribed and analysed thematically. Data from both sources were integrated to generate conclusions.

**Results:**

The domain score for basic amenities in both states was 48.8%; 47.0% in Anambra and 50.8% in Oyo state with 95% confidence interval [− 15.29, 7.56]. In Oyo, only half of the facilities (50%) had access to constant power supply compared to 72.7% in Anambra state. The overall general service readiness index for both states was 69.2% with Oyo state having a higher value (73.3%) compared to Anambra with 65.4% (*p* = 0.56). The domain score for availability of staff and TB guidelines was 57.1% for both states with 95% confidence interval [− 13.8, 14.4]. Indicators of this domain with very low values were staff training for the management of HIV and TB co-infection and training on MDR -TB. Almost half (47.6%) of the facilities experienced a stock out of TB drugs in the 3 months preceding the study. The overall tuberculosis-specific service readiness index for both states was 75%; this was higher in Oyo (76.5%) than Anambra state (73.6%) (*p* = 0.14). Qualitative data revealed areas of deficiencies for TB service delivery such as inadequate infrastructure, poor staffing, and gaps with continuing education on TB management.

**Conclusions:**

The weak health system remains a challenge and there must be concerted actions and funding by the government and donors to improve the TB healthcare systems.

## Background

Tuberculosis (TB) is a global health problem and a leading cause of deaths resulting in approximately 1.3 million deaths among HIV-negative individuals as well as 300, 000 additional deaths in people living with HIV and AIDS [1, 2]. According to the 2018 World Health Organization Global Tuberculosis Report, Nigeria has a TB incidence rate of 219 per 100,000 population while the estimated incidence of Multi-drug Resistant, rifampicin-resistant TB (MDR/RR-TB) was 12 per 100,000. Nigeria was listed as one of the top 20 countries with the highest incident TB cases among people living with HIV and in the general populations [2]. The national government through the Federal Ministry of Health responded to this challenge by setting three major targets for TB control in the country which are “*to detect at least 70% of all TB cases by 2020, achieve a treatment success rate of at least 90% for all new bacteriologically confirmed TB cases by 2020 and eliminate TB as a public health problem (<= 1/1,000,000 population) by 2050*” [3].

Globally, the WHO proposed the End TB Strategy which details interventions, objectives and targets for tuberculosis prevention and control [4]. The DOTS Strategy has been implemented with varying success in different parts of the world though most of the countries in sub-Saharan Africa including Nigeria have not recorded significant progress in the achievement of the global targets [1]. Factors attributed to this include the low priority and inadequate funding for TB prevention and control by national governments. This is reflected in the weak health systems characterised by poor infrastructure for TB care, insufficient human resources, poor diagnostic, and laboratory services [2, 5–8].

The successful implementation of the End TB strategy and sustainability interventions for TB control depends largely on the capacity of the general health systems within which TB services are delivered; this is succinctly captured as its “service readiness”. The term “readiness” is the level of preparedness and capacity of a health facility to provide holistic, quality, and comprehensive care to patients. The key indicators for readiness are the availability of trained staff, guidelines, infrastructure, medical commodities, essential drugs, and diagnostic capacity [9]. The WHO has developed the Service Availability and Readiness Assessment (SARA) tool which has standards for TB service readiness and countries are expected to utilise this to track the progress in health systems strengthening [9]. Several studies have been conducted in Nigeria to assess the health system, quality of TB care and management [7, 8, 10, 11]. However, there is a dearth of information on the general and TB-specific service readiness for the management of tuberculosis.

Oyo and Anambra, two states in Nigeria, are priority settings for TB control because they contribute significantly to the high TB burden in the country accounting for the highest prevalence in the South-west and South-east regions respectively [12]. Therefore, the objective of this study was to assess the service readiness for the delivery of TB services in the two states. Assessing the service readiness will guide interventions to improve the health system which is likely to have a synergistic effect on improving the quality of care for TB prevention and control thus contributing to the goal of the elimination of the disease [13].

## Methods

This was part of a large facility-based study which utilised the Donabedian’s framework to assess TB health care quality from three dimensions: the structure, process, and outcome [14]. A mixed-methods convergent parallel design was used to collect data from 13 and 12 Local Government Areas (LGAs) or districts in Anambra and Oyo states respectively. Specifically, data were collected using a semi-structured health facility assessment tool, an observational checklist and in-depth interview guides. Both the quantitative and qualitative data collection had equal weighting and occurred concurrently. This approach was adopted to aid the collection of different but complimentary data on TB service readiness in the study sites. This approach also has a potential to aid the triangulation of data and enrich the interpretation of the results. Data from both sources were analysed separately and results integrated to generate conclusions.

Structure encompasses the context in which care is delivered and assesses the potential capacity of the provider or institution to deliver quality health care. Process denotes all activities between patients and providers throughout the delivery of health care, and outcomes are measures of health i.e. the result of care on the health of the patient [15]. We present findings of the component on *“Structure”* of TB control program which reflects the potential capacity of the healthcare system to provide quality TB care.

### Study area and selection of TB facilities

As mentioned, Anambra and Oyo are the two states with the highest TB burden in the South-east and South-west regions respectively [12]. Based on the Nigerian 2006 census figures, Anambra state has a population of 4,182,032 and 21 LGAs while Oyo State has a population of 5,591,589 and 33 LGAs. Number of TB facilities were obtained from the State TB Focal Persons of the Ministries of Health in both states. In 2017 when the study was conducted, Anambra state had 62 TB facilities while Oyo had 53. The selection of the TB treatment facilities were based on four criteria: (i) DOT facilities supported by the Global Fund TB project (ii) Rural-urban representation (iii) ownership status of facility i.e. government or private facility and (iv) levels or tiers of care i.e. primary, secondary or tertiary health care centres. In Nigeria, the National Tuberculosis Leprosy Control Programme coordinates the training of TB staff through the development of curricula and guidelines and the conduct of training programmes in collaboration with the State Tuberculosis Leprosy Control Programme and donor organisations. Healthcare workers are trained during pre-service, in-service, continuing education and on-the-job mentoring.

### Training of research assistants and pretesting of tools

Prior to data collection, the research team conducted a three-day training for 10 research assistants per state; five males and five females who had postgraduate degrees in public health or the social sciences. The training focused on the objectives of the study, interview techniques, procedures for data collection and ethical issues. Thereafter the quantitative and qualitative research tools were pre-tested in two facilities in urban and rural DOT centres and revised as appropriate prior the conduct of the actual study.

### Quantitative component

The trained research assistants interviewed 42 TB staff using a semi-structured, interviewer-administered health facility assessment tool (additional file 1). The health facility assessment tool was adapted from the TB Service Availability and Readiness Assessment module of the Nigeria Health Facility Assessment Tool [16] and WHO SARA [17] and had questions on the availability and competency of staff involved in tuberculosis service delivery, availability and functionality of infrastructure, equipment, diagnostics, commodities, drugs and supplies for TB service delivery in primary, secondary and tertiary health facilities. A facility observational checklist was used to assess the infrastructure in each facility (additional file 2). This checklist was adapted from tools used in a previous study [7] and had questions on the waiting area, status of the examination room, drug storage area, sanitary facilities and infrastructure (floor, walls, roofs etc). The quantitative data was obtained from officers in charge of the TB facilities (focal persons) and the most senior health workers. These were selected as described below:

#### Sample size determination

The recommended formula by WHO SARA [17] for determining the sample size for health facilities was used. The sample size calculation took into cognisance the strata of interest and the formula is described as follows:

$$ \mathrm{n}=\Big[\left[\left(\mathrm{z}2\ast \mathrm{p}\ast \mathrm{q}\right)+\mathrm{ME}2\right] $$

$$ \left[\mathrm{ME}2+\mathrm{z}2\ast \mathrm{p}\ast \mathrm{q}/\mathrm{N}\right]\Big]\ast \mathrm{d} $$

Where

N = Total number of facilities in both states which is 115

z = the square of the normal deviate at the required confidence level (3.84 is the square of the normal deviate (1.96) needed to provide an estimate at the 95% level of confidence)

p = the proportion of facilities with the attribute of interest (50% was used since there is no estimate for TB service readiness from previous studies in Nigeria)

ME = margin of error (15% is generally used for SARA studies [17])

d = the design effect (assuming 1 [17])

The minimum sample size of facilities to be selected was 32 and this was increased to 42 to account for non-response.

#### Sampling procedure

A multistage sampling technique was used.

- Stage I: One state each with the highest TB prevalence (Oyo and Anambra) was selected from the South-west and South-east geopolitical zones of Nigeria.
- Stage II: The list of all LGAs and facilities providing TB care in the two states was obtained. This was stratified by ownership/management of facility classified as *public or private/faith-based*, level of the facility i.e. *primary, secondary, and tertiary* and location i.e. *urban, semi-urban and rural*. Facilities within each stratum were selected using simple random sampling.
- Stage III: Forty-two DOT PHC facilities were randomly selected from both states across 25 LGAs (13 and 12 in Anambra and Oyo states respectively). Tertiary and secondary health facilities were over-sampled in line with the SARA recommendations [17]. Sixteen public and private secondary health facilities (11 in Anambra and 6 in Oyo State) and three tertiary facilities were selected.
- Stage IV: In selected PHC facilities, the officer in charge of TB (focal persons) and the most senior health workers including medical officers, nurses, Community Health Officers (CHO), Community Health Extension Workers (CHEWs) where applicable, who were involved in TB care were interviewed. In each of the secondary and tertiary TB care facilities selected, the medical officers/consultants- in-charge, the most senior of these cadres of health care category (nurse, CHO, CHEW) was selected for the interview (In all, 42 staff were interviewed for the health facility assessment).

#### Measures of variables

The key dependent variables were the general and specific readiness of the health facilities for TB management. We assessed three domains for general service readiness which were basic amenities (power, water supply, sanitary facilities for clients, consulting room with privacy, communication equipment and computing facilities), basic equipment (adult and child weighing scales, thermometer) and infection prevention (safe disposal of sharps, infectious wastes and appropriate storage of sharp waste). The availability of these were categorised as “Yes” if available and “No” if not [9].

For TB service readiness, defined as the “the capacity of the facilities to provide care and treatment for TB”, we assessed three domains -*staffing and guidelines, diagnostics, medicines and commodities.* Staffing and guidelines have 8 indicators which are: guidelines for (i) diagnosis and treatment of TB, (ii) management of HIV and TB co-infection (iii) MDR-TB treatment including identification of need for referral (iv) TB infection control and the availability of at least one staff member providing services trained in the last 2 years on (v) TB diagnosis and treatment (vi) HIV and TB co-infection (vii) MDR-TB and (viii) TB infection control. The indicator for diagnostics are three - (i) availability of TB microscopy, (ii) HIV diagnostic capacity and (iii) system for the diagnosis of HIV among TB clients while the availability of first-line TB medications is the only indicator for medicines and commodities [9].

#### Explanatory variables

The explanatory variables were the ownership/management of facility classified as *public or private/faith-based* and the levels of the facility which were primary, secondary and tertiary. Facility location was categorised as urban, semi-urban and rural.

#### Data analysis

Prior to data entry, each of the administered instruments was checked in the field and reviewed for completeness. Data were cleaned and coded. A logic check was developed to minimize data entry errors. Statistical Package for Social Sciences (SPSS) Version 22 was used to analyse the data. Findings were summarised and presented in tables and figures.

The score for indicators “tracer items” for each of the three domains (basic amenities, equipment and infection prevention) were summed and expressed as percentages (“mean availability of tracer items as percentage within that domain”) to obtain the general service readiness and a similar approach was adopted for the three domains of the TB-specific service readiness. The mean of all the 3 domains for general and TB-specific service readiness were computed and expressed as general or TB specific service readiness index [9].

The results of the categorical variables were presented as frequencies and percentages and presented on tables while Anova or independent t-test was used to assess the association between the outcome variables i.e. *general or TB-specific readiness* and the explanatory variables. Statistical significance was set at P -value less than 0.05 at 95% Confidence Interval (CI).

### Qualitative component

In-depth interview guides (Additional file 3) were used to conduct key informant interviews (KIIs) with 58 TB staff (*2 State TB coordinators, 23 LGA TB supervisors and 33 TB focal persons*). The number of individuals interviewed reflects the recruitment of TB staff working in diverse settings *(ownership by public or private institutions, level of the facility* i.e. *primary and secondary and location* i.e. *urban, semi-urban and rural)* thus providing an opportunity to interview a diverse range of perspectives aimed at attaining data saturation. The respondents were purposively selected based on their roles in TB service delivery. The KII guides were used to explore factors influencing the quality of TB service delivery. The KII guides were adapted from tools used in a previous study [7] and had questions and probes on TB treatment guidelines and protocols, availability of skilled and trained TB staff, diagnostics and laboratory services in the facilities, health information system for TB service provision and reporting, drug supplies and logistics, challenges and recommendations for improved TB service provision.

 Trained research assistants and some of the researchers - MMO, AMA, DAA and GAA interviewed the TB Focal staff or designee using the guides. The trained research assistants had postgraduate degrees in public health or the social sciences while the researchers had postgraduate/doctorate degrees in medicine, public health, medical sociology and library and information sciences. The authors/researchers (*six males and three female*) are academic/research staff at the College of Medicine, University of Ibadan; Federal Medical Centre and Association for Reproductive and Family Health, Nigeria and each had over 15 years of experience in public health research and qualitative studies while the trained research assistants (*five males and five females*) were public health consultants. All the researchers are Nigerians who have deep understanding of the clinical/public health settings as well as the cultural context of the study sites. Prior the conduct of the interviews, the researchers had no contact with the interviewees. At the commencement of the study, the researchers wrote letters to the State TB Control Officers at the State Ministries of Health explaining the purpose of the study and requesting permission to interview the facility TB staff. After the request was approved, the State TB Control Officer informed the facility TB staff about the proposed study and provided their telephone details to the researchers. The researchers called the TB staff to schedule appointments for the interviews and they all agreed to participate in the study. The interviews were held face-to-face in offices within the DOT facilities which were free of distraction and noise and offered privacy. The interviews, which were recorded on digital recorders, lasted an average of 35 min per interview and were conducted concurrently with the quantitative data collection. The interviews were facilitated by a moderator and a note taker documented the process and interviewees’ verbal responses and non-verbal expressions. Prior to the interviews, informants were provided with detailed information on the objectives of the study and assurance of confidentiality, and permission to use digital voice recorder was made. Written informed consent was obtained from all the interviewees.

#### Data analysis

The audio-recordings were transcribed verbatim into word documents. The researchers read the transcripts to get acquainted with the data; subsequently, there was a detailed review which aided the development of the coding guide. The coding guide had codes such as “staffing”, “training”, “diagnostics” etc. which aligned with the questions/sections in the quantitative instrument. The researchers also discussed the interview transcripts prior to coding, points of agreement and disagreement were noted and resolved. Subsequently, the word files were uploaded into NVIVO version 10, coded by four data coders and analysed using thematic analysis. Two members of the research team independently coded 10% (*N* = 5) of the transcripts and the inter-coder reliability was 84% which was adequate. In addition, there was triangulation of the qualitative and quantitative data and the research team supervised the interviews to ensure the trustworthiness of the data. The themes were used to generate the narratives and verbatim expressions noted and presented. We used the Consolidated Criteria for Reporting Qualitative Research (COREQ) checklist to report the qualitative research.

##### Data integration and synthesis method

There was an integration of the data through merging of the data sets [18]. This helped deepen understanding of the status of service readiness in the facilities by providing a richer qualitative description, and the factors influencing TB care. Themes from both data sets were compared to identify areas of commonalities or differences. The data from both sources were integrated during final data interpretation through narrative description using the weaving approach which involved a presentation of both the qualitative and quantitative findings together by themes [18].

### Ethical considerations

The University of Ibadan/University College Hospital’s Ethical Review Committee reviewed and approved the protocol for the study prior to the commencement of data collection. Written informed consent was obtained from each study participant after information was provided on the background of the researchers and their affiliations, the nature and purpose of the study, the fact that data collected will be used for research only and that participation was voluntary. All information provided by research participants were kept confidential. To facilitate confidentiality, the research assistants were carefully selected and trained on ethical issues including field supervision and interviewer’s skills. The researchers ensured limited access to digital responses of the participants and removed identifiers in the electronic data set. In addition, the tool and consent form were translated and administered in Yoruba and Igbo, the local languages spoken in Oyo and Anambra, respectively. The sum of ₦ 500 (US1.50) was given to each of the respondents in appreciation of the time taken to be interviewed.

## Results

### Socio-demographic characteristics of the respondents

Quantitative data were obtained from 42 TB staff who completed the structured, interviewer administered health facility assessment tool. There were more respondents in Anambra (52.4%) than Oyo state (47.6%). Most of the respondents were females (66.7%), nurses (52.4%) and the duration of service in the TB facility were less than 1 year (4.8%), 1 to 4 years (45.2%), 5 to 9 years (35.7%) and 10 years and more (14.3%). Over a third worked in public health facilities (71.4%) compared to those in privately owned health facilities (28.6%). In addition, most worked in primary (54.8%) or secondary (38.1%) healthcare facilities which were in urban (52.4%), semi-urban (23.8%) or rural (23.8%) LGAs.

For the qualitative interviews in both states, there were two TB state focal persons (3.4%), 23 LGA TB Supervisors (39.7%) and 33 TB facility focal persons or designees (56.9%). Most of the interviewees were females (58.6%) and worked in public government owned facilities (72.4%). The duration of years providing TB services at the health facility were ≤ 5 year (50%), 6 to 10 years (43.1%) and 11 to 15 years (6.9%). Most worked in primary (44.8%) or secondary (24.1%) level facilities which were in urban (48.3%), rural (32.8%) or semi-urban LGAs (19%).

### DOTS facility profile by states

The profile of the facilities is summarized in Table 1. Twenty-three facilities were primary (10 in Anambra State and 13 in Oyo State), 16 secondary (10 in Anambra State and 6 in Oyo State) and three were tertiary (2 in Anambra State and 1 in Oyo State) healthcare facilities. Government-owned facilities accounted for 59.1% in Anambra state and 85% in Oyo state (see details in Table 1).

**Table 1:** DOT Facility Profile by States

Variable	Anambra n (%)	Oyo n (%)	Total n (%)
**Number of facilities visited**	22 (52.2)	20 (47.8)	42 (100)
**Number of LGAs**	13 (52.0)	12 (48.0)	25 (100)
**Tier of facility**			
Primary	10 (45.5)	13 (65.0)	23 (54.8)
Secondary	10 (45.5)	6 (30.0)	16 (38.1)
Tertiary	2 (9.0)	1 (5.0)	3 (7.1)
**Ownership of facility**			
Public	13 (59.1)	17 (85.0)	30 (71.4))
Private	4 (18.2)	1 (5.0)	5 (11.9)
Faith-Based	5 (22.7)	2 (10.0)	7 (16.7)
**Location of facility**			
Urban	8 (36.4)	14 (70)	22 (52.4)
Semi -urban	7 (31.8)	3 (15)	10 (23.8)
Rural	7 (31.8)	3 (15)	10 (23.8)

#### General service readiness at DOTS facilities

Table 2 presents the results for the three domains of general service readiness, *specifically basic amenities, basic equipment, and standard precautions for infection prevention*. The domain score for basic amenities in both states was 48.8%; 47.0% in Anambra and 50.8% in Oyo (95% CI, − 15.29, 7.56) In Oyo, only half of the facilities (50.0%) had access to constant power supply compared to 72.7% in Anambra. On the other hand, only 59.1% of facilities in Anambra had consulting rooms with privacy compared with 85.0% in Oyo. Availability of sanitation facilities was 63.6% and 80.0% in Anambra and Oyo respectively. Findings from the in-depth interviews provide further insights on the condition of infrastructure in TB facilities. The infrastructural inadequacies in both states are provided in the quotes below:

**Table 2:** General service readiness at DOTS facilities by states

Variable	Anambra n (%) *N* = 22	Oyo n (%) *N* = 20	Total n (%) *N* = 42	*P* value	95% CI
**Basic amenities**					
^**a**^**Improved water source**					
Yes	16 (72.7)	14 (70.0)	30 (71.4)		
**Electricity power supply**					
Yes	16 (72.7)	10 (50.0)	26 (61.9)		
^a^**Improved sanitation**					
Yes	14 (63.6)	16 (80.0)	30 (71.4)		
**Consulting room with privacy**					
Yes	13 (59.1)	17 (85.0)	30 (71.4)		
**Communication equipment**					
Yes	4 (18.2)	11 (55.0)	15 (35.7)		
**Computing facilities**					
Yes	1 (4.5)	2 (10.0)	3 (7.1)		
**Domain score (mean availability of items as percent) [Mean (SE)]**	47 (4.0)	50.8 (3.9)	48.8 (2.8)	0.33	−15.29, 7.56
**Basic equipment**					
**Weighing scale**					
Yes	15 (68.2)	17 (85.0)	32 (76.2)		
**Thermometer**					
Yes	22 (100)	20 (100)	42 (100)		
**Domain score (mean availability of items as percent) [Mean (SE)]**	84.1 (5.1)	92.5 (4.1)	88.1 (3.3)	0.01	−21.6, 4.79
**Infection Prevention**					
Appropriate storage of sharp waste	14 (63.6)	18 (90)	32 (76.2)		
Safe disposal of sharps	17 (77.3)	15 (75.0)	32 (76.2)		
Appropriate storage of infectious waste	14 (63.6)	18 (90)	32 (76.2)		
Safe disposal of infectious waste	17 (77.3)	15 (75.0)	32 (76.2)		
Soap and running water available	13 (59.1)	11 (55.0)	24 (57.1)		
Latex gloves available	14 (63.6)	13 (65.0)	27 (64.3)		
**Domain score (mean availability of items as percent) [Mean (SE)]**	65.2 (5.4)	76.7 (4.1)	70.6 (3.5)	0.60	−25.3, 2.31
**Availability of structural problems in the health facility**					
Roof	9 (40.9)	8 (40.0)	17 (40.5)		
Ceiling	11 (50.0)	13 (65.0)	24 (57.1)		
Wall	7 (31.8)	6 (30.0)	13 (31.0)		
Floors	7 (31.8)	5 (25.0)	12 (28.6)		
Painting	9 (40.9)	6 (30.0)	15 (35.7)		
Plumbing	8 (36.4)	6 (40.0)	14 (33.3)		
Drainage	4 (18.2)	5 (25.0)	9 (21.4)		

^a^based on standards for improved water sources and sanitation promoted by UNICEF

*“Erratic power supply is the major problem we face and we lack a generator that would have replaced the lack of direct power supply. The laboratory results that should be out within 24 hours can take days and even weeks … patients most times would have to keep calling to know when to come for their results”(Female_TB_FP_Pry_Public_Rural _Oyo state)*

*“ you know the [*Xpert MTB/RIF*] is electricity driven, the erratic power supply is another problem that is affecting the optimal functioning of this Xpert MTB/RIF machine so that is one of the challenges we are facing” (Oyo State TB Focal Person)*

In Anambra state, the gaps with the provision of basic amenities was expressed in the quote below:

“ *You have seen it, we don't have latrines here, we don't have chairs, and the government just gives the manpower. Even this curtain was done by us to tidy up this area” (TB_ FP _Pry _Public_Urban _Anambra state)*

About half of the facilities in Anambra and Oyo had problems with the roof. Ceiling problems were the highest reported structural building challenges in 57.0% of facilities, followed by roof (40.5%) and painting (35.7%). In Anambra and Oyo, ceiling problems accounted for the most common challenge, (50.0%) and (65.0%) respectively (Table 2).

*“We have a waiting area that is not functioning because of the roof that is bad so we don’t always have space where we can gather patients to give them health education and all that is so concerning, we need help, then for the waste we don’t have any place to dispose them, then water supply we need that also … …* .*then ventilation here is not good enough because we share facilities with a primary health centre (for maternal and child health) and you can see that it is choked up and there is no movement of air” (Female_TB_ Facility Focal Person _Pry_ Public_Urban _ Oyo state)*

With regards to basic equipment, the domain score for both states was 88.1%; 84.1% and 92.5% in Anambra and Oyo respectively (95% CI: − 21.6, 4.79). Availability of weighing scale was lower in facilities in Anambra (68.2%) compared to Oyo (85.0%). The overall domain score for infection prevention in both states was 70.6%; this was slightly higher in Oyo 76.7% compared to Anambra 65.2% (95% CI: − 25.3, 2.31). Specifically, both states have poor availability of soap and running water, and gloves − 59.1% and 55.0% and 63.6% and 65% in Anambra and Oyo states respectively (Details in Table 2).

#### General service readiness index by health facility characteristics

**Fig. 1:**
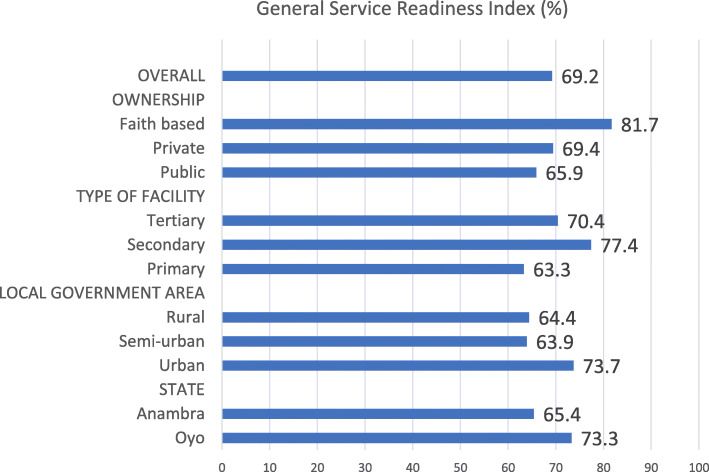
General service readiness index by health facility characteristics

*The general service readiness index provides a summary status of basic amenities, basic equipment, and standard precautions for infection prevention in the states. The data in* Fig. 1 showed that the overall general service readiness index for both states 69.2% with Oyo state having a higher score (73.3%) than Anambra (65.4%) (*p* = 0.56). The general service readiness index varied by facility ownership/management; faith-based health facilities had a higher value (81.7%) than public/government owned (65.9%) (*p* = 0.03). This reflects the variations in the general service readiness with faith-based and private health facilities having higher values. Facilities in urban areas had higher general service readiness index compared to those in semi-urban or rural areas (*p* = 0.09). Primary health facilities had lower general service readiness index compared to secondary or tertiary healthcare facilities (*p* = 0.01) (Fig. 1).

#### Tuberculosis specific service readiness at DOTS facilities

##### Human resources, staff training and availability of guidelines

The proportion of human resources for health and TB service provision varied in both states. Over a fifth, (22.7%) of facilities in Anambra and a tenth in Oyo had only one staff. Over a third of (35.0%) of facilities in Oyo had 2 health workers while in Anambra, a fifth (22.7%) had a similar number. Almost a third, (31.8%) of facilities in Anambra had five and above personnel as against 10% in Oyo state. Six facilities had Medical Officers in Anambra as against two in Oyo (Table 3). Some of the TB facilities have inadequate staff as indicated in the quote below:

**Table 3:** Total number of health workers by cadre at the DOTS centres

Variable	Anambra n (%) *N* = 22	Oyo n (%) *N* = 20	Total n (%) *N* = 42
**Total number of health workers**			
1	5 (22.7)	2 (10.0)	7 (16.7)
2	5 (22.7)	7 (35.0)	12 (28.6)
3	3 (13.6)	6 (30.0)	9 (21.4)
4	2 (9.1)	3 (15.0)	5 (11.9)
≥ 5	7 (31.8)	2 (10.0)	9 (21.5)
**Number of staff by cadre**			
Medical Officers	6 (27.2)	2 (10.0)	8 (19.0)
Community Health Officers	2 (9.1)	4 (13.3)	6 (14.3)
Public Health Nurse	1 (4.5)	1 (7.7)	2 (4.8)
Midwives	6 (27.2)	–	6 (14.3)
Staff Nurses	8 (36.3)	5 (25.0)	13 (40.0)
Nurses/midwives	4 (18.2)	2 (10.0)	6 (14.3)
Senior Community Health Extension Workers	4 (18.2)	8 (61.5)	15 (35.7)
Junior Community Health Extension Workers	3 (13.6)	2 (15.4)	5 (11.9)
Environmental Officers	1 (4.5)	–	1 (2.4)
Pharmacy technicians	2 (9.1)	–	2 (4.8)
Medical Records Officers	1 (4.5)	–	1 (2.4)
Nutrition Officers	–		–
Laboratory technicians	11 (50.0)	2 (10.0)	13 (40.0)
Health Assistants	2 (9.0)	6 (30.0)	8 (19.0)

*“ … … ..we don’t have adequate staff … … … … . so am the one recording, am the one taking samples except the laboratory scientist that is helping us to diagnose on the AFB, the microscopy process and everything I handle, I will take their samples to the lab, I will document the result of the lab, then if I need to place them on drugs, I am the one that will do that, that is the reason why I said we don’t have enough staff”* *(Male_TB_FP_ Pry_Public_Urban _PHC_1_Oyo State).*

According to the State TB focal persons, a key factor responsible for inadequate staff at the government health facilities is the retirement of trained staff and the failure of the state and local governments to recruit adequate health workers to replace the retired staff. Another factor expressed is the inability of the State TB Control programme to influence staff distribution at all levels, especially the primary healthcare. The inadequate human resources for TB facilities is not peculiar to the public/government-owned health facilities; a similar situation was reported in the private facilities as reflected in the quote below which indicated the need for more health workers because they provide 24-h services: The need for more staff in the private health facilities may be pressing because majority provide 24-h services for most healthcare needs:

*We also need manpower because we run 24 hours’ services* (TB FP_Sec Fac_Private_Rural_1, Anambra state).

The domain score for availability of trained staff and TB guidelines was 57.1% for both states; 57.3% in Anambra and 57% in Oyo (95%CI: − 13.8, 14.4). Indicators of this domain with very low values were staff training for the management of HIV and TB co-infection (*27.3% in Anambra and 0% in Oyo state*) and training on MDR -TB in the last 2 years (18.2% in Anambra and 30% in Oyo state) (see details in Table 4). The WHO SARA tool assesses staff training on various aspects of TB care in the last 2 years preceding the survey and this largely focuses on continuing education on TB care. According to a respondent in Anambra state, staff working at the TB clinics are healthcare professionals who had undergone modules on TB care as part of the curricula and requirement for qualification as skilled healthcare workers. However, there were gaps with continuing education and professional development on TB management. For instance, as illustrated in the quote below, some staff had not been trained on TB care though one had participated in a training on MDR-TB. This reflects the training gaps as well as the unstructured and unsystematic approach to continuing education programmes on TB care in the state.

*“ … … .. all of them are trained nurses and nurse midwives [trained on TB care as part of the requirement for qualifications as skilled healthcare workers], they are all trained staff, but they have not been trained on TB management [continuing professional development and training on TB care]. That is the only problem. The other person, one of them have been trained on MDR just MDR but not on TB care. The other two have not been trained at all”* (*TB FP_Public_Sec_Urban_, Anambra state)*

**Table 4:** Tuberculosis specific service readiness at DOTS facilities by states

Variables	Anambra n (%)	Oyo n (%)	Total n (%)	*p*-value	95% CI
**Staff and Guidelines**					
Guidelines for diagnosis and treatment of TB					
-Yes	17 (77.3)	18 (90)	35 (83.3)		
Guidelines for management of HIV and TB co-infection					
-Yes	17 (77.3)	18 (90)	35 (83.3)		
Guidelines for TB infection control					
-Yes	17 (77.3)	18 (90)	35 (83.3)		
Staff trained for TB diagnosis and treatments					
-Yes	18 (81.8)	16 (80)	34 (81)		
Staff trained for HIV and TB co-infection					
- Yes	6 (27.3)	0 (0)	6 (14.3)		
Staff trained for MDR-TB					
- Yes	4 (18.2)	6 (30)	10 (23.8)		
Staff trained for hiv					
- Yes	17 (77.3)	18 (90)	35 (83.3)		
**Domain score (mean availability of items as percent) [Mean (SE)]**	57.3 (5.5)	57 (4.2)	57.1 (3.4)	0.11	−13.8, 14.4
**Diagnostics**					
Availability of TB microscopy					
- Yes	14 (63.6)	13 (65.0)	27 (64.3)		
System for the diagnosis of HIV among TB clients					
- Yes	22 (100)	20 (100)	42 (100)		
**Domain score (mean availability of items as percent) [Mean (SE)]**	81.8 (5.3)	82.5 (5.5)	82.1 (3.7)	0.86	−16.0, 14.6
**Medicines and commodities**					
Availability of first-line TB medications					
- Yes	18 (91.8)	18 (90)	36 (85.7%)		
**Domain score (mean availability of items as percent) [Mean (SE)]**	81.8 (8.4)	90 (6.7)	85.7 (5.5)	0.13	−30.4, 14.1

The unsystematic approach to training needs assessment and plan was also documented in Oyo state. According to a respondent, the trainings were infrequent and unpredictable as illustrated below:

*“ … … … It [the training] is not frequent, it is unpredictable. They will just call us whenever they feel that we need to be updated, they will just call us maybe from the state or from the federal” (Male_TB_FP_Pry_Public_Semi-urban_ Oyo state)*

Continuing education and training gaps were also reported in Oyo state and the State TB Focal person provided more insight into the gaps in continuing education and training on TB care. Specifically, he expressed that a significant proportion of the staff had not undergone formal continuing education programme on TB care due to funding constraints. The strategy adopted is the transfer of basic skills during the onsite monitoring visits and this finding was corroborated by one of the healthcare workers as expressed below:

“*the TBLS Local Government Supervisor usually organises an [onsite] update on the latest development, for the staff working with her so everybody is informed*” *(Female_TB_FP_Pry_Public_Semi-urban _ Oyo state).*

##### Diagnostics

The domain score for diagnostics was 82.1% for both states. Only two third of facilities in both states (63.6% in Anambra and 65% in Oyo states) had a TB microscopy (Table 4). All the DOTS facilities had a system for the diagnosis of HIV among TB patients. Findings further revealed that there were functional laboratory facilities in 75 and 65% of DOTS facilities in Anambra and Oyo respectively. X-ray and Xpert MTB/RIF were available and functional in 30% and 25% of DOTS centres in Anambra and 30% and 15% of facilities in Oyo respectively**.**

The quotes below illustrate the conditions of the laboratories in the two states.

*“ … we do not have a laboratory, we use to take our samples to the general hospital and at times it takes ten days for the results to come out and it has been giving us concerns” (Female TBLS, Rural, Oyo state).*

*“ … .but the problem is that we don’t have enough Xpert MTB/RIF material so those are the issues and even the ones that we have presently in the country they are just 4 modules machine that can only handle 4 samples within 2 hours … .. those are some of the challenges that we are facing presently, and you know generally in Nigeria we are faced with the problem of low case finding” (State TB Focal Person)*

Even when there was a laboratory facility, equipment such as Xpert MTB/RIF and microscope were either not available at all or non-functional. The following illustrates the challenges in this regard:

*‘It is not good because our gene expert is not good. Only two modules are working others are not functioning. So sometimes we go to a private hospital where they have gene expert, or we go to Enugu [a city] for gene expert but they are promising to repair … Another is manpower. The building is not well ventilated, the door is spoilt, and our health is in danger and we are still doing the work (TB_FP_Secondary_ Anambra state)*

Some respondents in Anambra also expressed the gaps with regards to the functionality of laboratory facilities and the turn-around time for results as stated in the quotes below:

“*at times they [the general hospital where we take our sputum samples for testing] are overwhelmed with work, so at times when we go with sputum it is delayed for a week, that of last week we are yet to collect it … … Yes and it may not be up to two weeks sometimes, it may be early*” (M*ale_TB_FP_Pry_Public_Rural_ Oyo state)*

*‘The lab results are not coming out as it supposed to. Like the one we did at the general hospital it took two days'. (Male_FP_Private_Sec_Anambra State)*

However, some private facilities had a contrary opinion as they reported that it only takes a duration of 24 h in their facilities:

‘*Sometimes it takes 24hours because if someone brings sputum this morning, you can run it and they will receive it [the result] tomorrow morning*’ *(Male_FP_Private_Sec_Anambra State).*

*‘That same day. They will not wait to get it. The moment they bring the sample they will surely get it that same day’ (Female_FP_Private_Sec_Anambra State)*

##### Medicines and commodities

**Fig. 2:**
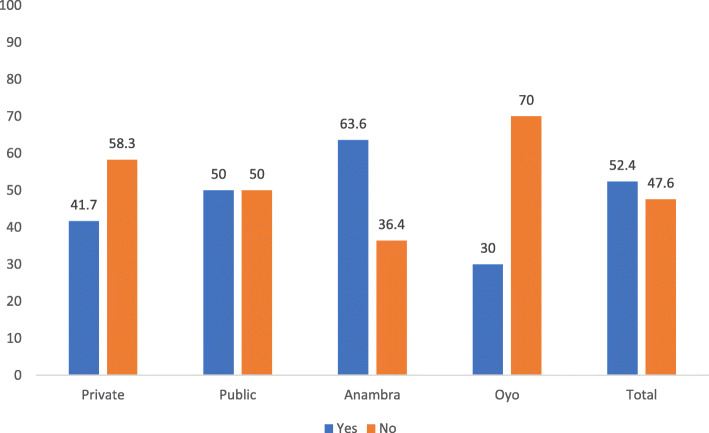
Experience of stock out of TB drugs in treatment facilities

The domain score for medicines and commodities was 85.7% for both states (Table 4). Ninety percent of facilities in both states (91.8% in Anambra and 90% in Oyo) had first-line TB medications at the time of the assessment. However, findings further revealed that, 3 months preceding the study, 47.6% of the facilities in both states had experienced a stock out of TB drugs. Most (63.6%) of the facilities in Anambra state had experienced a stock out of TB drugs; however, the problem was minimal in Oyo state where only 30.0% experienced stock out of essential drugs. Furthermore, 41.7% of private facilities compared to 50% of public facilities had experienced TB drug stock out (Fig. 2)

.

According to the TB State Focal persons, TB drug stock outs occur only occasionally because there is an efficient logistics management system. However sometimes, there may be delays with the importation and distribution of TB drugs which invariably results in stock outs. The quote below illustrates this point:

*Well the issue (stock out) is due to the lag in the distribution of drugs … ..usually, we don’t have TB drug stock out but presently, the drugs we have are inadequate and we are expecting another set … … .what they (Federal Ministry of Health) told us is that there is delay in the importation and distribution of the drugs (Male_State TB Focal Person)*

In addition, facilities have devised means of addressing the stock out such as borrowing drugs from other facilities which is returned when they receive their re-supply of drugs.

Quotes from the in-depth interviews buttress this finding as stated below:

*Now when drugs are not enough, the patients may be seventy-three and we may be given drugs for forty-five. [So] we give the drugs of a patient to another patient till we are supplied, we order for drugs from the state and return the drug of the patient back … We manage by taking the drug of a patient and administering it out to another patient but before two weeks they supply us another drug* … … *(Male_TB_FP_Pry_Public_Urban _Oyo state)*

… … *in fact, if there is [delay] in bringing our drug sometimes, we normally borrow from other facilities to return immediately our drug is brought (Female_TB_FP_Pry_Public_Rural _ Oyo state)*

#### Tuberculosis-specific service readiness index by health facility characteristics

**Fig. 3:**
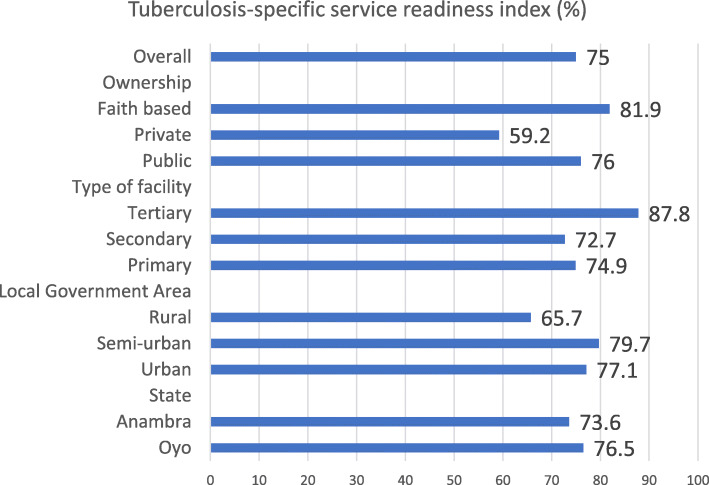
Tuberculosis specific service readiness index by health facility characteristics

The overall tuberculosis-specific service readiness index for the states was 75%; this was higher in Oyo state (*p* = 0.14) tertiary hospitals (0.34), health facilities owned by faith-based institutions (*p* = 0.07) and those located in semi-urban local government areas = 0.10) (Fig. 3).

## Discussion

This study has revealed that overall, there are several gaps in the general and TB-specific service readiness of TB facilities in the two states surveyed and these vary by geographical location, type and ownership of the health facilities. Some of the facilities assessed did not have adequate infrastructure to support the provision of quality services. The problems include poor power supply, inadequate water and soap, poor privacy during consultations, among others. Other researchers have reported similar findings on poor infrastructural facilities which undermine the provision of quality services and effective infection prevention measures [7, 19]. These pose an occupational risk to health workers as well as contribute to nosocomial infections in patients. Sub-standard infection control measures contribute to poor treatment, prognosis, disease progression with potential transmission risks to health workers, their families, and the development of drug-resistant TB strains [19, 20]. Upgrading the DOT centre through provision of basic amenities such as good toilet facilities, regular supply of clean and potable water, improved ventilation and constant power supply should be a priority of government and the funding partners.

There is also concern about poor staffing; almost half (45%) of the facilities in both states had only one or two staff. The qualitative data illustrates the dire situation with staffing. Similar poor staffing challenges have been reported in Nigeria and other countries with a high TB burden [10, 21, 22]. This has implication for the quality of service provided; for example, studies in Lesotho and Uganda identified poor staffing situation as a major barrier to the application of infection control guidelines and the effective implementation of measures such as screening for people with a cough, health education and timely sputum examination [21, 23]. This has also been identified as a barrier to the successful implementation of DOTS in Nigeria [10, 11]. There is, therefore, an urgent need to improve the staffing situation in all the DOTS centres in order to increase efficiency and effectiveness of the services rendered and reduce heavy workload of the few available staff who are vulnerable to errors, fatigue and burnout. It is also important that there should be fair and equitable distribution of all the cadre of staff, which is part of the best practice in TB treatment and control programming. Every DOT centre should have a fair representation of all the staff ranging from community health extension worker to laboratory scientist, pharmacist, and a supervising medical officer. This will ensure not just the number of different categories of staff are available in each facility but also enhance the quality of the services provided.

Another area of concern is the finding that only a quarter of facilities in Anambra and a fifth in Oyo had the Xpert MTB/RIF and that some DOT centres did not have a laboratory. The importance of the Xpert MTB/RIF is underscored by the fact that it is needed for the initial diagnostic test for TB. The WHO has recommended that the Xpert MTB/RIF should replace sputum smear microscopy because it is faster and more accurate [1]. The non-availability of laboratory facilities and the Xpert MTB/RIF are major causes of delay in providing prompt results of sputum tests which are necessary to confirm the diagnosis before treatment can commence. There is, therefore, a need to close the diagnostic gap by ensuring facilities are equipped with the Xpert MTB/RIF [2] and supported with necessary infrastructure including regular electricity supply.

Another important finding from this study that may undermine the quality of service is the experience of TB drugs stock out which was found to be a more serious problem in Anambra than Oyo state. However, the challenge of stock-out is not peculiar to DOT facilities but a common problem reflecting the poor health system in Nigeria as a whole [24]. Stock-out contributes to loss of confidence in the health care system and a major cause of treatment interruption. Some TB patients respond to this challenge by buying their medications from pharmacy shops, unfortunately, medicines purchased in these outlets may be fake and sub-standard resulting in treatment failure. To mitigate this challenge, the Nigeria’s Federal Ministry of Health must explore feasible interventions to reduce TB drug stock out. A potential intervention which is underway in South Africa is the Visibility & Analytics Network (VAN) operating model which has been adopted by this country’s national health agency to coordinate medication supply chain management. Key features of this bundle of interventions is the use of data across the medicine supply chain by specialized supply chain planners to enhance drug cost effectiveness and availability; analytical processes which optimizes ordering recommendations and decisions and continuously improve processes coupled with a network that links the facilities to the national level and stipulate their roles supported by IT systems for enhance communications [25].

It is commendable that virtually all the health workers interviewed in both Anambra and Oyo states confirmed that they had received pre-service training on TB prevention, treatment and management. Training is a major incentive that boosts the morale of staff to provide good quality of care for patients. However, we observed some deficit with respect to continuing education because some of the health workers reported that they had not received training on some important components of TB management such as co-infection with HIV and multi-drug resistant TB. This has implication because studies have linked inadequate training on TB care to poor adherence to the national treatment guidelines [26, 27] and treatment outcomes. A feasible approach to bridge this gap is the use of distance learning platforms especially certified global training programmes such as the CDC’s online resource for TB training and education and the WHO global resource and training course on “Implementing the WHO End TB strategy” [28]. These courses are hosted online and offer great potentials for continuing TB education considering the reduced cost for hosting on-site training programmes and the potential to reach a larger audience [28]. Another approach which holds promise is the use of Mobile Instant Messaging which has been tested and found effective for the transfer of skills and the professional development of nurses in Nigeria [29, 30].

Our study has limitations and strengths. On limitation, the data were obtained from two TB high burden states which are in two out of the six geopolitical regions in Nigeria and this limits the generalizability of our findings. However, the study utilized a mixed methods approach and ensured the data obtained reflected the range of health facilities in Nigeria thus deepening our understanding and perspectives on readiness for TB service delivery in different healthcare settings. Another strength is the triangulation of data from multiple sources, and emphasis on the need for regular internal and external audits of TB services in Nigeria. Outcomes would remain the same or get poorer if processes remain the same.

The findings of this study has brought to fore, the need for improved funding of TB healthcare services. It is a well-known fact that TB services in Nigeria are largely donor-driven [31] and the program runs mainly on the benevolence of the donor agencies and countries. There is obviously a limit to what the donors can do. Government at all levels in Nigeria must commit a commensurate amount of their resources to TB programming if the country is to meet its targets for TB control.

## Conclusions

The weak healthcare systems remain a major barrier for addressing tuberculosis care and control in Nigeria. Stronger health systems are key to achieving the global targets for TB control. Government and other donors must prioritise funding for health system strengthening to achieve the global and national targets which are: *the detection of all forms of TB cases, a treatment success rate and elimination of TB as a public health problem.* The interventions should focus on improving the quality and quantity of human resources for TB care, equipping DOT centres, provision of infrastructural facilities to support care and regular supply of TB drugs and other medical supplies.

## Supplementary information

**Additional file 1.** Health Facility Assessment Tool.

**Additional file 2.** Facility Observational Checklist.

**Additional file 3.** Interview Guides.

## Data Availability

The datasets are available upon request to the corresponding author. This can only be used for non-commercial purposes while maintaining participants’ confidentiality.

## Abbreviations

CHO: Community Health Officer
CHEW: Community Health Extension Workers
JCHEW: Junior Community Health Extension Workers
DOTs: Directly Observed Treatment
FP: Focal Person
TB: Tuberculosis
MDR/RR-TB: Multi-drug Resistant, Rifampicin-Resistant Tuberculosis
Pry: Primary
Sec: Secondary
KII: Key Informant Interview
