# Supplementary material for: General and tuberculosis-specific service readiness in two states in Nigeria
Source: BMC Health Serv Res. 2020 Aug 26;20:792. doi: 10.1186/s12913-020-05626-3 (PMC7448989; doi:10.1186/s12913-020-05626-3)
Supplement: Supplementary file 1 — Additional file 1. Health Facility Assessment Tool. [file 12913_2020_5626_MOESM1_ESM.docx]

***QUALITY OF TUBERCULOSIS SERVICE DELIVERY AND TREATMENT OUTCOMES IN NIGERIA***

**HEALTH FACILITY ASSESSMENT TOOL**

### Introduction

Greeting. My name is ________________________________ I am here on behalf of the College of Medicine, University of Ibadan, Nigeria. The goal of this study is to evaluate the quality of TB service delivery and outcomes by cadres of health workers and types of health facilities in two states in Nigeria.Your participation in this survey will be highly appreciated.Your participation is entirely voluntary and your decision whether or not to participate will involve no penalty or loss of benefits.

Remember:

- There are no right and wrong answers, this is not an examination.
- Please answer all the questions as honestly and accurately as you can — this is very important and will help improve the delivery of TB care in this facility.

If you have further questions or concerns, please contact the undersigned

**Professor Ademola Ajuwon (Principal Investigator), Department of Health Promotion and Education, Faculty of Public Health, College of Medicine, University of Ibadan, Nigeria. Email:ajajuwon@yahoo.com; Phone number: 08034892561**

**Will you like to participate in the survey? 1. Yes [ ] 2. No [ ]**

**NOTE TO INTERVIEWER:** *This tool must be completed by the facility head or designee*

**DEMOGRAPHIC INFORMATION OF PERSONNEL INTERVIEWED**

1. Participant’s title/designation ……………………………………………………………………
2. Primary Responsibilities .…………………………………………….………………………………………………………………………………………………………………………………………………………………………………………………………………………………………………………………………
3. Respondent’s phone number ……………………………………………………………
4. How long have you worked at this facility? ………………….**.**......................................
5. What is the designation of the person responsible for managing TB and leprosy services at this facility?
6. Medical Doctor
7. Nurse
8. Pharmacy Technician
9. Pharmacy Assistant
10. Pharmacist
11. Store Officer
12. Medical Attendant
13. Assistant Medical Officer
14. Laboratory Scientist
15. Laboratory Technician
16. Laboratory Assistant
17. Other

| **Section 1: Identification Particulars** | |
| --- | --- |
| **Q0.1** | State: |
| **Q0.2** | Town: |
| **Q0.3** | L.G.A: |
| **Q0.4** | Ward: |

| **Section 2: Geographical information system** | | | | | | | | | | | | | |
| --- | --- | --- | --- | --- | --- | --- | --- | --- | --- | --- | --- | --- | --- |
| **1. Identification** | | | | | | | | | | | | | |
| **Item** | |  | | | | | | | |  | | | |
| Facility Name: | |  |  |  |  |  |  |  |  |  |  |  |  |
| Facility type (tick where appropriate) | | **Tertiary PHC** | | **Secondary** | | **Primaryy** | | **Public** | | | **Private/ FBO** | | **Other** |
|  |  |  | |  | |  | |  | | |  | |  |
| Address of facility | |  | | | | | | | | | | | |
| **2. Position** | | | | | | | | | | | | | |
| **Elevation/Altitude/Height (Meter)** | | | | | | | | | | | | | |
|  | **Degrees** | | | | **Decimal Degrees** | | | | | | | | |
| **Latitude N** |  | |  | |  | |  | |  | | |  | |
| **Longitude E** |  | |  | |  | |  | |  | | |  | |
| **Level of accuracy**  **(meters)** |  | | | |  | | | | | | | | |

| **Section 3: Infrastructure of Health facilities** | | | | | | | |
| --- | --- | --- | --- | --- | --- | --- | --- |
| **No.** | **Questions** | | | **Yes** | | **No** | **Skip to** |
| **Q3.1** | When was the facility built? | | | Year:……….  Don’t know……- | | |  |
| **Q3.2** | Who owns the health facility?  ***(Tick only one)*** | Government.....................................................  Rented..............................................................  Private..............................................................  Community......................................................  Religious organization..................................... | | | | |  |
| **Q3.3** | What is the source of water available at the health centre?  ***Tick all that apply*** | Bore hole..........................................................  Uncovered well................................................  Covered well....................................................  Stream..............................................................  Water tanker.....................................................  Harvested rain water........................................  Piped water.......................................................  None.................................................................  Others (specify)___________________________ | | | | |  |
| **Q3.4** | Is there electricity power supply at the facility? | | | **No** | | **Yes** | **If no, go to Q3.6** |
|  |  |  |  |  | |  |  |
| **Q3.5** | Indicate source(s) of power supply  ***Tick all that apply*** | National grid.....................................................  Solar power.......................................................  Generator..........................................................  Combination with Inverter...............................  Others (specify)__________________________ | | | | |  |
| **Q3.6** | Toilet facilities available  (***Tick all that apply)*** | None …………….............................................  Flush latrine (WC)............................................  Ventilated improved pit latrine........................  Open pit latrine.................................................  Hanging toilet...................................................  Open Defeacation.............................................  Others (specify) __________________________ | | | | |  |
| **Q3.7** | Is the health facility fenced? | | | **No** | **Yes** | |  |
|  |  |  |  |  |  | |  |
| **Q3.8** | How many security personnel are there in the health facility? ***(write number)*** | | |  | | |  |
| **Q3.9** | Is there any major road leading to the facility? | | |  |  | | **If no, go to Q3.11** |
| **Q3.10** | If yes, what type?  **(*Tick all that apply)*** | | Tarred road......................................................  Foot path..........................................................  Untarred road...................................................  Others (specify)_________________________ | | | |  |
| **Q3.11** | Is there any structural problem in the building?  *Roof*  *Ceiling*  *Wall*  *Floors*  *Painting*  *Plumbing*  *Drainage* | | | **No** | | **Yes** | **Specify** |
|  |  |  |  |  | |  |  |

| **Section 4: Human Resources for Health and TB service Provision** | | | | | | |
| --- | --- | --- | --- | --- | --- | --- |
| *Now we would like to ask questions related to staffing in the TB center* | | | | | | |
| **Q4.1** | In total, how many health workers are there in the TB center? **(*Please write the total number)*** | |  | |  | |
| **Q4.2** | Can you tell us the number of staff by cadre? | **Cadre** | | **Number** |  | |
|  |  | Medical Officer | |  |  |  |
|  |  | Community Health Officer | |  |  |  |
|  |  | Public Health Nurse | |  |  |  |
|  |  | Midwives | |  |  |  |
|  |  | Nurse | |  |  |  |
|  |  | Nurse/Midwives (All Categories) | |  |  |  |
|  |  | SCHEWs (All Categories) | |  |  |  |
|  |  | JCHEWs (All Categories) | |  |  |  |
|  |  | Environmental Health Officers | |  |  |  |
|  |  | Pharmacy Technicians | |  |  |  |
|  |  | Medical Records Officers | |  |  |  |
|  |  | Nutrition Officers | |  |  |  |
|  |  | Laboratory Technician | |  |  |  |
|  |  | Others (Specify)_____________________ | |  |  |  |
|  | *Now we would like to ask questions related to staffing in the TB DOTs center at your facility* | | | | | |
| **Q4.3** | In total, how ***many health workers in the TB DOTs center have been trained on TB service provision*** **in the last 2 years**? **(*Please write the total number).*** | | |  | |  |
|  | Can you tell us the number of staff by cadre?  List the numbers by cadre | **Cadre** | | **Number** | |  |
|  |  | Medical Officer | |  | |  |
|  |  | Community Health Officer | |  | |  |
|  |  | Public Health Nurse | |  | |  |
|  |  | Midwives | |  | |  |
|  |  | Nurse | |  | |  |
|  |  | Nurse/Midwives (All Categories) | |  | |  |
|  |  | SCHEWs (All Categories) | |  | |  |
|  |  | JCHEWs (All Categories) | |  | |  |
|  |  | Environmental Health Officers | |  | |  |
|  |  | Pharmacy Technicians | |  | |  |
|  |  | Medical Records Officers | |  | |  |
|  |  | Nutrition Officers | |  | |  |
|  |  | Laboratory Technician | |  | |  |
|  |  | Others (Specify)_____________________ | |  | |  |

| **Section 5:Managerial Capacity** | | | | | | | | | | | | | | | | | | | | | | | | | | | | | | | | |  |  |  |
| --- | --- | --- | --- | --- | --- | --- | --- | --- | --- | --- | --- | --- | --- | --- | --- | --- | --- | --- | --- | --- | --- | --- | --- | --- | --- | --- | --- | --- | --- | --- | --- | --- | --- | --- | --- |
| **Team building** | | | | | | | | | | | | | | | | | | | | | | | | | | | | | | | | |  |  |  |
| **Q5.1** | How often do facility staff hold meetings on management of the TB DOTS center?  ***(check one only)*** | | | | Never.................................................................  \Weekly..............................................................  Monthly.............................................................  Quarterly...........................................................  Annually............................................................  Others................................................................ | | | | | | | | | | | | | | | | | | | | | | | | | |  | |  |  |  |
| **Q5.2** | If the Officer in Charge is away from duty who takes responsibility for running the facility? (designation) | | | | | | | | | | | | | | | | | | | | | | | | | | | | | |  | |  |  |  |
| **Supervision** | | | | | | | | | | | | | | | | | | | | | | | | | | | | | | | | |  |  |  |
| **No.** | **Questions** | | | | | | | | | | | | | | | | | | **No** | | **Yes and sighted** | | | | | | **Yes but not sighted** | | | | | | | **Skip to** |  |
| **Q5.3** | Have you received any supervision visit by the National or State Tuberculosis and Leprosy Control Programme? (Check visitors’ book, if necessary, and site copies of the supervisory checklists/ feedback reports.) | | | | | | | | | | | | | | | | | |  | |  | | | | | |  | | | | | | | **If no, go to Q5.10** |  |
| **Q5.4** | If yes, how often do they conduct supervisory visits?  *Specify* | | | | | | | | | | | | | | | | | | Monthly...........  Quarterly..........  Annually..........  Never................  Not specific ......  Others ……………….. | | | | | | | | | | | | | | |  |  |
| **Q5.5** | When was the most recent supervision visit by the National or State Tuberculosis and Leprosy Control Programme? ***Indicate the date*** | | | | | | | | | | | | | | | | | |  | | | | | | | | | | | | | | |  |  |
| **Q5.6** | How many supervisory visits have been conducted in the last 3 months? **(*Please write the total number)*** | | | | | | | | | | | | | | | | | |  | | | | | | | | | | | | | | |  |  |
| **Q5.7** | Who do they supervise/? (List the designations/units below)  ***Write in the space below*: units supervised** | | | | | | | | | | | | | | | | | | | | | | | | | | | | | | | | |  |  |
| **Q5.8** | Is there a checklist for supervision? | | | | | | | | | | | | | | | | | | **No** | | | | **Yes** | | | | | | **Don’t know** | | | | |  |  |
|  |  |  |  |  |  |  |  |  |  |  |  |  |  |  |  |  |  |  |  | | | |  | | | | | |  | | | | |  |  |
| **Q5.9** | Do they provide feed back to the person(s) /units supervised? | | | | | | | | | | | | | | | | | |  | | | |  | | | | | |  | | | | |  |  |
| **Q5.10** | Have you received any training on supportive supervision in the last one year? | | | | | | | | | | | | | | | | | |  | | | |  | | | | | |  | | | | |  |  |
| **Section 6: Planning and budgeting** | | | | | | | | | | | | | | | | | | | | | | | | | | | | | | | | |  |  |  |
| **No.** | **Questions** | | | | | **No** | | | | | | | | | | | | | | | | | **Yes** | | | | | | | | | | |  |  |
| **Q6.1** | Does the TB DOTs centre have a workplan? | | | | |  | | | | | | | | | | | | | | | | |  | | | | | | | | | | |  |  |
| **Q6.2** | Who developed it? (specify) | | | | | In collaboration with Health workers...............  In collaboration with Community....................  In collaboration with LGA...............................  The PHC coordinator/Hospital Head................  In collaboration with others (Specify) ___________ | | | | | | | | | | | | | | | | | | | | | | | | | | | |  |  |
| **Q6.2** | Does the TB DOTs centre have a budget? | | | | | **No** | | | | | | | | | | | | | | | | | **Yes** | | | | | | | | | | |  |  |
|  |  |  |  |  |  |  | | | | | | | | | | | | | | | | |  | | | | | | | | | | |  |  |
| **Q6.3** | Who developed it? (specify) | | | | In collaboration with Health workers...............  In collaboration with Community....................  In collaboration with LGA...............................  The PHC coordinator/Hospital Head................  In collaboration with others (Specify) ___________ | | | | | | | | | | | | | | | | | | | | | | | | | | | | |  |  |
|  |  | | | | | | | | | | | | | | | | | | **No** | | | | **Yes** | | | | | | | | | | |  |  |
| **Q6.4** | Have you received training on micro-planning? | | | | | | | | | | | | | | | | | |  | | | |  | | | | | | | | | | | **If no, go to Q6.6** |  |
| **Q6.5** | If yes, when? | | | | | | | | | | | | | | | | | | dd/ mm/yyyy | | | | | | | | | | | | | | |  |  |
| **Q6.6** | Does the TB DOTs center /facility have a catchment area? | | | | | | | | | | | | | | | | | |  | | | |  | | | | | | | | | | | **If no, go to Q7.1** |  |
| **Q6.7** | If yes, estimate the target population. | | | | | | | | | | | | | | | | | |  | | | | | | | | | | | | | | |  |  |
| **Section 7: Resource mobilisation** | | | | | | | | | | | | | | | | | | | | | | | | | | | | | | | | |  |  |  |
| **No.** | **Questions** | | | | | | | | | | | | | | | | | | **No** | | | | **Yes** | | | | | **Skip to** | | | | |  |  |  |
| **Q7.1** | Does the health facility mobilise resources outside statutory allocation? | | | | | | | | | | | | | | | | | |  | | | |  | | | | | **If no, go to Q8.1** | | | | |  |  |  |
| **Q7.2** | If yes, state other sources of support/resources (***Write in the space below)*** | | | | | | | | | | | | | | | | | | | | | | | | | | |  | | | | |  |  |  |
| **Section 8: Service Delivery (*Anchored primarily on service provision using minimum health care package)*** | | | | | | | | | | | | | | | | | | | | | | | | | | | | | | | | |  |  |  |
| **No.** | **Questions** | | | | | | | | | | | | | | | | | | **No** | | | | **Yes** | | | | |  | | | | |  |  |  |
| **Q8.1** | Is there a laboratory at this health facility where TB tests are performed | | | | | | | | | | | | | | | | | |  | | | |  | | | | |  | | | | |  |  |  |
| **Q8.2** | If no laboratory is available at this facility, how are patients diagnosed for TB  ***(Tick all that applies)*** | | | | | | | | | | | | | | | | | | (i)Patients are sent to the laboratory at the state hospital  (ii)Patient’s sputum/blood samples are sent to the private hospital  (iii)Others (*Specify)* | | | | | | | | | | | | | |  |  |  |
|  |  | | | | | | | | | | | | | | | | | | **No** | | | | **Yes** | | | | | **Skip to** | | | | |  |  |  |
| **Q8.3** | Does this facility have a Standard Operating Procedure (a document) on TB service delivery? | | | | | | | | | | | | | | | | | |  | | | |  | | | | | **If no, go to Q8.5** | | | | |  |  |  |
| **Q8.4** | Do all health workers in this facility make use of TB Standard Operating Procedure for TB ***(Ask for a copy)*** | | | | | | | | | | | | | | | | | |  | | | |  | | | | |  | | | | |  |  |  |
| **Q8.5** | Does this TB DOTs center provide the following services? | | | | | | | | | | | | | | | | | | | | | | | | | | | | | | | |  |  |  |
|  |  | | | | | | | **No** | | | **Yes** | | | If so, how frequently are these services provided? | | | | | | | | | | | | | | | | | | |  |  |  |
|  |  |  |  |  |  |  |  |  |  |  |  |  |  | **Rarely** | | | | | | | | | | | **Sometimes** | | | | | **Regular** | | |  |  |  |
|  | Communication on TB | | | | | | |  | |  | | | |  | | | | | | | | | | |  | | | | |  | | |  |  |  |
|  | IEC campaign on TB | | | | | | |  | |  | | | |  | | | | | | | | | | |  | | | | |  | | |  |  |  |
|  | Community outreach programmes on TB | | | | | | |  | |  | | | |  | | | | | | | | | | |  | | | | |  | | |  |  |  |
| **Q8.6** | Does the health facility provide TB outreach services | | | | | | | | | | | | | | | | | | | | | | | | **Yes** | | | **No** | |  | | |  |  |  |
|  | *Follow Up* | | | | | | | | | | | | | | | | | | | | | | | |  | | |  | | **If no, go to Q8.8** | | |  |  |  |
|  | *Home Visit* | | | | | | | | | | | | | | | | | | | | | | | |  | | |  | |  |  |  |  |  |  |
|  | *Phone Calls* | | | | | | | | | | | | | | | | | | | | | | | |  | | |  | |  |  |  |  |  |  |
| **Q8.7** | If yes, indicate how often | | | | | | | | **Rarely** | | | | | | **Sometimes** | | | | | | | | | | **Regular** | | | | |  | | |  |  |  |
|  | *Follow Up* | | | | | | | |  | | | | | |  | | | | | | | | | |  | | | | |  |  |  |  |  |  |
|  | *Home Visit* | | | | | | | |  | | | | | |  | | | | | | | | | |  | | | | |  |  |  |  |  |  |
|  | *Phone Calls* | | | | | | | |  | | | | | |  | | | | | | | | | |  | | | | |  |  |  |  |  |  |
| **Q8.8** | Have some health workers in the DOTS center/unit received training on provision of TB services? | | | | | | | | | | | | | | | | | | **No** | | | | | **Yes** | | | | | | **If no, go to Q9.1** | | |  |  |  |
|  |  |  |  |  |  |  |  |  |  |  |  |  |  |  |  |  |  |  |  | | | | |  | | | | | |  |  |  |  |  |  |
| **Q8.9** | *If yes, indicate trainingreceived and date****.*** | | | ***Type of training*** | | | ***No. trained*** | | | | | ***In the past 6 months*** | | | | | ***In the past 1 year*** | | | | | | | ***>1-2 years*** | | | | **>2-5 years** | | | | |  |  |  |
|  |  |  |  | TB diagnosis and treatments | | |  | | | | |  | | | | |  | | | | | | |  | | | |  | | | | |  |  |  |
|  |  |  |  | HIV and TB co-infection | | |  | | | | |  | | | | |  | | | | | | |  | | | |  | | | | |  |  |  |
|  |  |  |  | MDR-TB | | |  | | | | |  | | | | |  | | | | | | |  | | | |  | | | | |  |  |  |
|  |  |  |  | TB infection control | | |  | | | | |  | | | | |  | | | | | | |  | | | |  | | | | |  |  |  |
|  |  |  |  | Others | | |  | | | | |  | | | | |  | | | | | | |  | | | |  | | | | |  |  |  |
| **Section 9: Strengthen of NHMIS for programme monitoring and management in the Health Facility** | | | | | | | | | | | | | | | | | | | | | | | | | | | | | | | | |  |  |  |
| **No.** | | **Questions** | | | | | | | | | | | | | | | | **No** | | | | **Yes** | | | | | | **Skip to** | | | | |  |  |  |
| **Q9.1** | | | Do you use NHMIS forms in this facility? | | | | | | | | | | | | | | | |  | | | |  | | | | | **If no, go to Q9.3** | | | | |  |  |  |
| **Q9.2** | | | If yes which of the following guidelines/forms are available and adequate in your health facility/ward? | | | | | | | | | | | | | | | | | | | | | | | | | | | | | |  |  |  |
|  |  |  |  | | | | | | | | | | **Available** | | | | | | | | | | | | | **Adequate *(probe for ease of use)*** | | | | | | |  |  |  |
|  |  |  |  |  |  |  |  |  |  |  |  |  | **No** | | | **Yes** | | | | **N/A** | | | | | | **No** | | **Yes**  **nNo** | | | | **No N/A** |  | | |
|  |  |  | NTLCP workers manual | | | | | | | | | |  | | |  | | | |  | | | | | |  | |  | | | |  |  | | |
|  |  |  | Guidelines for diagnosis and treatment of TB | | | | | | | | | |  | | |  | | | |  | | | | | |  | |  | | | |  |  | | |
|  |  |  | Guidelines for management of HIV and TB co-infection | | | | | | | | | |  | | |  | | | |  | | | | | |  | |  | | | |  |  |  |  |
|  |  |  | Guidelines for TB infection control | | | | | | | | | |  | | |  | | | |  | | | | | |  | |  | | | |  |  |  |  |
|  |  |  | TB unit registry | | | | | | | | | |  | | |  | | | |  | | | | | |  | |  | | | |  |  |  |  |
|  |  |  | TB referral & transfer form | | | | | | | | | |  | | |  | | | |  | | | | | |  | |  | | | |  |  |  |  |
|  |  |  | TB sputum examination request form | | | | | | | | | |  | | |  | | | |  | | | | | |  | |  | | | |  |  |  |  |
|  |  |  | Quarterly case finding, treatment outcome and other TB control activity report form | | | | | | | | | |  | | |  | | | |  | | | | | |  | |  | | | |  |  |  |  |
|  |  |  | TB flip chart in consulting room | | | | | | | | | |  | | |  | | | |  | | | | | |  | |  | | | |  |  |  |  |
|  |  |  | TB posters in waiting area | | | | | | | | | |  | | |  | | | |  | | | | | |  | |  | | | |  |  |  |  |
|  |  |  | Functional weighing scale for adults | | | | | | | | | |  | | |  | | | |  | | | | | |  | |  | | | |  |  |  |  |
|  |  |  | Functional weighing scale for children | | | | | | | | | |  | | |  | | | |  | | | | | |  | |  | | | |  |  |  |  |
|  |  |  | Thermometer available | | | | | | | | | |  | | |  | | | |  | | | | | |  | |  | | | | |  |  |  |

| **Q8.3** | Is there a designated Health /Medical Records Officer? | | | | | **No** | | | | | | **Yes** | | | | **Skip to** |
| --- | --- | --- | --- | --- | --- | --- | --- | --- | --- | --- | --- | --- | --- | --- | --- | --- |
| **Q9.3** | Is there a designated Health /Medical Records Officer? | | | | |  | | | | | |  | | | | **If no, go to Q9.9** |
| **Q9.4** | Is this staff a qualified Health/Medical Records Officer? | | | | |  | | | | | |  | | | |  |
| **Q9.5** | Has this staff been trained in NHMIS? | | | | |  | | | | | |  | | | |  |
| **Q9.6** | If yes when? | | | | | **____/_____/_____**  dd/ mm/yyyy | | | | | | | | | |  |
| **Q9.7** | Is this designated staff involved in routine M & E activities? | | | | | **No** | | | | **Yes** | | | | | | **If yes, go to Q9.10** |
|  |  |  |  |  |  |  | | | |  | | | | | |  |
| **Q9.8** | If No, give reasons (***Write in the space below)***  (a)  (b)  (c) | | | | | | | | | | | | | | |  |
| **Q9.9** | Are the data generated from routine M & E activities available at the facility? | | | | | **No** | | | **Yes** | | | | | | |  |
|  |  |  |  |  |  |  | | |  | | | | | | |  |
| **Q9.10** | Are the data analyzed and reviewed at the facility? | | | | |  | | |  | | | | | | |  |
| **Q9.11** | Are data submitted to the LGA/State TB and Leprosy Control Programme? | | | | |  | | |  | | | | | | | **If no, go to Q9.15** |
| **Q9.12** | If yes, how often?  ***Tick one that applies*** | | Weekly.............................................................  Bi-weekly.........................................................  Monthly............................................................  Quarterly.......................................................... | | | | | | | | | | | | |  |
| **Q9.13** | Has there been any feed back from the LGA/ State TB and Leprosy Control Programme from the data submitted? | | | | | **Never** | | | | **Rarely** | | | | | | **Frequently** |
|  |  |  |  |  |  |  | | | |  | | | | | |  |
| **Q9.14** | How was the feedback information from the LGA used? **(*Write in the space provided below)*** | | | | | | | | | | | | | | |  |
| **Q9.15** | Rate (**by circling**) the extent to which the following activities are provided in your facility | | **Activity** | | | **Not at all** | | | **Partially provided** | | | | | | | **Fully Provided** |
|  |  |  | Routine data collection | | | 0 | | | 1 | | | | | | | 2 |
|  |  |  | Data storage | | | 0 | | | 1 | | | | | | | 2 |
|  |  |  | Data Entry | | | 0 | | | 1 | | | | | | | 2 |
|  |  |  | Data Cleaning | | | 0 | | | 1 | | | | | | | 2 |
|  |  |  | Data analysis | | | 0 | | | 1 | | | | | | | 2 |
|  |  |  | Dissemination and information sharing | | | 0 | | | 1 | | | | | | | 2 |
| **Q9.16** | Is there a dedicated office for Medical Records in this facility | | | | | **No** | | | | **Yes** | | | | | |  |
|  |  |  |  |  |  |  | | | |  | | | | | |  |
| **Q9.17** | How many employees work in this Records Unit/Department? | | Female: __________________  Male: ____________________  Total: _____________________ | | | | | | | | | | | | |  |
| **Q9.18** | Which of the following equipment do you have dedicated to data processing, information and communication? | | | | | | | | | | | | | | |  |
|  | **EQUIPMENT** | | **AVAILABLE**  **AND FUNCTIONAL/ADEQUATE**  **(Indicate Number)** | | **AVAILABLE**  **NOT FUNCTIONAL/NOT ADEQUATE**  **(Indicate Number)** | | | | | | **NOT**  **AVAILABLE**  **(Mark X)** | | | | |  |
|  | Calculator | |  | |  | | | | | |  | | | | |  |
|  | Mobile Phone/ Telephones | |  | |  | | | | | |  | | | | |  |
|  | Laptop | |  | |  | | | | | |  | | | | |  |
|  | Stationeries *(Pencils Erasers, Rulers, Cardboard and markers)* | |  | |  | | | | | |  | | | | |  |
|  | Desktop computer with necessary accessories | |  | |  | | | | | |  | | | | |  |
|  | Others (specify) ………..…………. | |  | |  | | | | | |  | | | | |  |
| **Q9.19** | In what mode is data displayed at the facility?  ***Tick all that apply*** | | | Reports...........................................................  Tables………………………………….....….  Charts..............................................................  Graphs.............................................................  Others (specify) | | | | | | | | | | |  |  |
| **Section 10: Logistics system and infrastructure** | | | | | | | | | | | | | | | |  |
| **Q10.1** | | How do you receive TB medical supplies and commodities?  ***Tick all that apply*** | | From donors...................................................  ***(Specify donor)***………………………………  Purchased by facility.......................................  From Government...........................................  No delivery of supplies................................... | | | | | | | | | | |  |  |
| **Q10.2** | | Does the LGA//STBLCP deliver the TB medical supplies to you “PUSH”? | | | | | **No** | | | | | | **Yes** | |  |  |
|  |  |  |  |  |  |  |  | | | | | |  | |  |  |
| **Q10.3** | | Do you go to the LGA//STBLCP to collect the medical supplies “PULL”? | | | | |  | | | | | |  | |  |  |
| **Q10.4** | | How do you determine your requirement of medical supplies?  ***Tick all that apply*** | | Based on consumption....................................  Demographic data...........................................  Storage capacity..............................................  Service statistics..............................................  Others (specify) __________________________ | | | | | | | | | | |  |  |
| **Q10.5** | | Who bears the cost of delivery of medical supplies and commodities?  ***Tick all that apply*** | | LGA................................................................  STBLCP..........................................................  SMOH..............................................................  Partners/NGOs.................................................Others (specify)_ | | | | | | | | | | |  |  |
| **Q10.6** | | Does the DOTs centre/health facility have a store for medical supplies and commodities? | | | | | | **No** | | | | | | **Yes** | **Skip to** |  |
|  |  |  |  |  |  |  |  |  | | | | | |  |  |  |
| **Q10.7** | | Does the DOTs centre/ health facility have a solar refrigerator? | | | | | |  | | | | | |  |  |  |
| **Q10.8** | | Do you use safety boxes for storing sharps? | | | | | |  | | | | | |  |  |  |
| **Q10.9** | | How are infectious medical wastes disposed of at this facility?  ***Tick all that apply*** | | Burn & Bury...................................................  Incinerator.......................................................  Open dumping .............….........................…...  Others (specify)___________________________ | | | | | | | | | | |  |  |
|  |  | How are sharps disposed of at this facility?  ***Tick all that apply*** | | Burn & Bury...................................................  Incinerator.......................................................  Open dumping .............….........................…...  Others (specify)___________________________ | | | | | | | | | | |  |  |
| **Q10.10** | | Please sight and describe the waste disposal site (***Write in the space below)*** | | | | | | | | | | | | |  |  |
| **Q10.11** | | Is the essential drugs list displayed in your health facility? | | | | **No** | | | **Yes** | | | | | |  |  |
|  |  |  |  |  |  |  | | |  | | | | | |  |  |
| **Q10.12** | | Do you keep ledgers for the essential drugs?  ***Tick yes only if sighted*** | | | |  | | |  | | | | | |  |  |
| **Q10.13** | | Do you have bin cards? ***Tick yes only if sighted*** | | | |  | | |  | | | | | |  |  |
| **Q10.14** | | Do you use Stores Issued Vouchers and Stores Received Vouchers (SIV and SRV respectively)? | | | |  | | |  | | | | | |  |  |
| **Q10.15** | | Do you use stock ledger to manage TB health commodities in the facility | | | |  | | |  | | | | | |  |  |
| **Q10.16** | | Have you experienced stock-outs of essential TB drugs in the last three months? ***Please check records***  ***Tick yes only if both vouchers are being used*** | | | |  | | |  | | | | | |  |  |
| **Q10.17** | | If yes, please list the type of essential TB drugs that were stocked-out. (***Write in the space below:*** | | | | | | | | | | | | |  |  |
| **Q10.18** | | How do you replenish your drug supply?  ***Tick all that apply*** | | From LGA........................................................  From STBLCP................................................  Ad hoc..............................................................  At open market.................................................  Others (specify)___________________________ | | | | | | | | | | |  |  |

**Q10.20:** Please complete the table 1 on stock availability below. Circle which drug or medical supply (if any) that is currently stocked out.

**Table 1: Stock Availability**

| **Product** | **Unit** | **Used at this facility? (Y/N)** | **Physical verification of product (Y/N)** | **Stock out today? (Y/N)** | **Stock card available? (Y/N)** |  |
| --- | --- | --- | --- | --- | --- | --- |
| Rifampicin 150mg /Isonizid100mg /Pyrazinamide400mg (RHZ) | pill |  |  |  |  |  |
| Ethambutol/Isoniazide (EH) | pill |  |  |  |  |  |
| Streptomycin (STM) | pill |  |  |  |  |  |
| Complete drugs for each patient | pill |  |  |  |  |  |
| STOP TB drug kit | pill |  |  |  |  |  |
| Storage area for drugs *(comment freely)* |  | | | | |  |
| Laboratory supplies  *(comment freely)* |  |  |  |  |  |  |

**Q10.21 Challenges encountered in TB Service delivery: ………………………………………………………………………………………………………………………………………………………………………………………………………………………………**

**Q10.22 Recommendations for improved TB Service delivery:**

**………………………………………………………………………………………………………………**

**………………………………………………………………………………………………………………**

**………………………………………………………………………………………………………………**

**Thank the interviewee for participation!**
