# Supplementary material for: General and tuberculosis-specific service readiness in two states in Nigeria
Source: BMC Health Serv Res. 2020 Aug 26;20:792. doi: 10.1186/s12913-020-05626-3 (PMC7448989; doi:10.1186/s12913-020-05626-3)
Supplement: Supplementary file 2 — Additional file 2. Facility Observational Checklist. [file 12913_2020_5626_MOESM2_ESM.docx]

***QUALITY OF TUBERCULOSIS SERVICE DELIVERY AND TREATMENT OUTCOMES IN NIGERIA***

***Checklist for the observation of facilities, equipment and infrastructure***

| INTERVIEWER OBSERVATIONS | | | | | | | | | | | | |
| --- | --- | --- | --- | --- | --- | --- | --- | --- | --- | --- | --- | --- |
|  |  | | **YES** | | **NO** | **SATISFACTORY** | | | **FAIR** | | **UNSATISFACTORY** | |
|  | ***Patient flow and organization (i.e. Patients are attended to how they arrive)*** | |  | |  |  | | |  | |  | |
|  | ***Vital signs/statistics of patients taken*** | |  | |  |  | | |  | |  | |
|  | ***Observations on the records system (e.g. the condition, quality, accessibility, security and organization of the records.)***  if 3 of the 5 variables above are good, tick “satisfactory”; if 2 are good, tick “fair”, less than 2 is unsatisfactory | |  | |  |  | | |  | |  | |
|  | ***Health education on TB control and personal hygiene*** | |  | |  |  | | |  | |  | |
|  | ***Attitudes of providers towards TB Care and Service Provision (greet clients, politeness; develop rapport with client(s) etc).*** | |  | |  |  | | |  | |  | |
|  | ***Recommendations for improved TB service delivery*** | |  | | | | | | | | | |
| **INTERVIEWER OBSERVATIONS** | | | | | | | | | | | | |
|  | | | | **Adequate** | | | **Not adequate** | | | **Comments** | | |
|  | | ***Waiting area*** | |  | | |  | | |  | | |
| i | | Space | |  | | |  | | |  | | |
| ii | | Cross Ventilation | |  | | |  | | |  | | |
| iii | | Protection from direct sunlight | |  | | |  | | |  | | |
|  | | ***Consulting room*** | | Available (Functional/Adequate = 1;  Non-functional/Not adequate = 0) | | | Non-available | | | Comments | | |
| i | | Privacy | |  | | |  | | |  | | |
| ii | | Water for washing hands | |  | | |  | | |  | | |
| iii | | Hand gloves | |  | | |  | | |  | | |
| vi | | Extractor fans | |  | | |  | | |  | | |
|  | | ***State of Maintenance of facilities*** | | Yes | | | No | | | Comments | | |
| i | | ***Floor and walls*** | |  | | |  | | |  | | |
| Ii | | Cracks | |  | | |  | | |  | | |
| iii | | Holes | |  | | |  | | |  | | |
|  | | ***Cleanliness*** | | Yes | | | No | | | Comments | | |
| i | | No litter on the floor | |  | | |  | | |  | | |
| ii | | No stains on the floor | |  | | |  | | |  | | |
| iii | | Absence of cobwebs and dust | |  | | |  | | |  | | |
|  | | ***Toilet facility*** | | Yes | | | No | | | Comments | | |
| **i** | | Toilet facility available | |  | | |  | | |  | | |
| ii | | Faeces littering toilet | |  | | |  | | |  | | |
| **iii** | | Foul odour | |  | | |  | | |  | | |
|  | | ***Diagnostics space*** | | **Available** (Functional = 1; Non-functional = 0) | | | **Non-available** | | | **Comments** | | |
| i | | Laboratory | |  | | |  | | |  | | |
| ii | | TB microscopY | |  | | |  | | |  | | |
| iii | | Air filter masks | |  | | |  | | |  | | |
| iv | | X ray machine | |  | | |  | | |  | | |
| v | | Gene Xpert machine | |  | | |  | | |  | | |
| vi | | Sputum cups | |  | | |  | | |  | | |
| vii | | HIV Test Kits | |  | | |  | | |  | | |
| viii | | Hand gloves | |  | | |  | | |  | | |
| Ix | | Water for hand washing (specify whether tap or in a container)  *(If available and adequate = 1; Not adequate = 0)* | |  | | |  | | |  | | |
| x | | Foul odour (No=1, Yes=0) | |  | | |  | | |  | | |
|  | | **Availability of materials** | | Yes (Good condition/functional = 1; Poor condition/non-functional = 0) | | |  | No | | | |  |
|  | | TB unit registry | |  | | |  |  | | | |  |
|  | | TB referral and transfer form | |  | | |  |  | | | |  |
|  | | TB sputum examination request form | |  | | |  |  | | | |  |
|  | | Quarterly case finding, treatment outcome and other TB control activity report form | |  | | |  |  | | | |  |
|  | | TB flip chart in consulting room | |  | | |  |  | | | |  |
|  | | TB posters in waiting area | |  | | |  |  | | | |  |
|  | | Functional weighing scale | |  | | |  |  | | | |  |
|  | | **Anti-TB drugs** | | **Yes** ( ≥3 months stock = 2; < 3 months stock = 1; Expired = 0) | | | | **No** | | | | |
| i | | Rifampicin/Isonizid/  Pyrazinamide (RHZ) | |  | | | |  | | | | |
| ii | | Ethambutol/Isoniazide (EH) | |  | | | |  | | | | |
| iii | | Streptomycin (STM) | |  | | | |  | | | | |
| iv | | Stop TB drug kit | |  | | | |  | | | | |
| iv | | Storage area for drugs | | **Yes** | | | | **No** | | | | |
|  |  |  |  |  | | | |  | | | | |
| v | | ***Please note any general observations you have that are not addressed in this survey.*** | |  | | | | | | | | |
