# Supplementary material for: General and tuberculosis-specific service readiness in two states in Nigeria
Source: BMC Health Serv Res. 2020 Aug 26;20:792. doi: 10.1186/s12913-020-05626-3 (PMC7448989; doi:10.1186/s12913-020-05626-3)
Supplement: Supplementary file 3 — Additional file 3. Interview Guides. [file 12913_2020_5626_MOESM3_ESM.docx]

***QUALITY OF TUBERCULOSIS SERVICE DELIVERY AND TREATMENT OUTCOMES IN NIGERIA***

**KEY INFORMANT INTERVIEW FOR TB FOCAL PERSONS AT THE STATE**

**Introduction**

I am here on behalf of the College of Medicine, University of Ibadan, Nigeria. The goal of this study is to evaluate the quality of TB service delivery and outcomes by cadres of health workers and types of health facilities in the two Nigerian states.Your participation is entirely voluntary and your decision whether or not to participate will involve no penalty or loss of benefits.

Remember:

- There are no right and wrong answers, this is not an examination.
- Please answer all the questions as honestly and accurately as you can — this is very important and help improve the delivery of TB care in this state.

If you have further questions or concerns, please contact the undersigned

**Professor Ademola Ajuwon (Principal Investigator), Department of Health Promotion and Education, Faculty of Public Health, College of Medicine, University of Ibadan, Nigeria. Email:ajajuwon@yahoo.com; Phone number: 08034892561**

**Note to Interviewer: *Share the study information sheet and request for signed consent***

**Demographics, TAKE NOTES** (identifying information to be kept separate from interview transcripts)

Just to confirm that I have your details right…..

1. Participant’s name & organization and email/ contact details (fill in beforehand if possible):
2. Participant’s title/designation and primary responsibilities:
3. What year did you start working in this organization? What year did you start in this particular position?

**REQUEST TO TURN ON RECORDERS AT THIS POINT IN THE INTERVIEW**

| **S.No.** | **Questions** | **Probes** |
| --- | --- | --- |
|  | Comment freely on the prevalence of TB among different age groups and gender in the last 5 years in your state | - *If increasing in the last 5 years, probe for reasons why* - *If decreasing in the last 5 years, probe for reasons why* - *If the prevalence has been stable in the last 5 years, probe for reasons why* - *Request for documented evidence( service statistics/records/reports)* |
|  | Can you explain how TB services are provided in your state and its influence on client satisfaction and treatment outcomes? | - *Approach for TB service provision* - *Client load* - *Client Satisfaction with quality of care* - *Schedules for community mobilization for early TB screening* - *Overall coordination and frequency of community outreach programmes* - *Effect of Community level interventions/outreach programmes on TB service utilization* - *Mechanism to solicit patient feedback* - *Adequacy and functionality of infrastructural facilities* - *Annual Performance Targets for TB care and service provision* |
|  | Which of the Tuberculosis treatment guidelines and protocols are you aware of?  To what extent have these been utilized by Health workers to guide TB service provision? | - *Availability and use of the following treatment guidelines*   *(Adult TB; Pediatric TB; MDR/XDR; TB/* *HIV co- infection; Infection prevention)*   - *When last were the treatment guidelines updated* - *Update/refresher training for health providers on the use of the guidelines* - *Extent of distribution and use at facilities* - *Potential barriers to provider adherence to the use of the guidelines* - *MDR TB notification systems; infection control policies* |
|  | In your views, does the state have adequate skilled staff for TB care and service provision? | - *Adequacy of staff by cadres* - *Frequency of training/refresher training and type of training ( i.e. pediatric TB treatment, adult TB treatment, MDR TB management, management and supervisory skills course, TB managers course, laboratory, infection prevention and control and community activities).* - *Supervision and monitoring systems*. |
|  | Can you explain the extent to which the resources for TB diagnostic and laboratory services are available in your state. | - *Availability of trained Laboratory technician on the use of recommended TB diagnostic test* - *Laboratory logistics and supplies* - *Turn-around times for lab results -* *sputum microscopy* |
|  | Please describe the adequacy of the National Health Information System for Tuberculosis care and service provision? | Probe for   - *Availability and actual use of registers* - *Extent of submission of data reporting tools to the next level and frequency of written of oral feedback from national level* - *Frequency of Data audit/assessment at facilities and STATE levels* - *Data quality issues* |
|  | Can you describe the drug and supplies logistics system for your state and gaps or strengths with regards to its effective management | Probe for   - *Availability of anti TB drugs* - *Standardized protocol for forecasting TB drugs* - *Mechanisms for forecasting, procurement, and distribution of drugs;* - *Supervisory mechanism for forecasting, procurement, and drug distribution* - *Episodes and duration of stock out in the last 6 months and mitigation efforts* |
|  | What are the challenges hindering TB delivery services? | - *Barriers to quality TB service provision -Probe for case detection and treatment* - *Probe for challenges at health facility and community levels?* |
|  | What would you recommend for improved TB service provision? | Probe:  *Staffing, Drug and Supplies, Health Information System, Infrastructural Facilities, Health financing etc*  *Ways to improve patients’ adherence to treatment, Improve Case detection and DOTS coverage* |

**Thank the interviewee for participation!**

***QUALITY OF TUBERCULOSIS SERVICE DELIVERY AND TREATMENT OUTCOMES IN NIGERIA***

**KEY INFORMANT INTERVIEW FOR TB FOCAL PERSONS AT THE LGAs**

**Introduction**

I am here on behalf of the College of Medicine, University of Ibadan, Nigeria. The goal of this study is to evaluate the quality of TB service delivery and outcomes by cadres of health workers and types of health facilities in the two Nigerian states.Your participation is entirely voluntary and your decision whether or not to participate will involve no penalty or loss of benefits.

Remember:

- There are no right and wrong answers, this is not an examination.
- Please answer all the questions as honestly and accurately as you can — this is very important and help improve the delivery of TB care in this state.

If you have further questions or concerns, please contact the undersigned

**Professor Ademola Ajuwon (Principal Investigator), Department of Health Promotion and Education, Faculty of Public Health, College of Medicine, University of Ibadan, Nigeria. Email:ajajuwon@yahoo.com; Phone number: 08034892561**

**Note to Interviewer: *Share the study information sheet and request for signed consent***

**Demographics, TAKE NOTES** (identifying information to be kept separate from interview transcripts)

Just to confirm that I have your details right…..

1. Participant’s name & organization and email/ contact details (fill in beforehand if possible):
2. Participant’s title/designation and primary responsibilities:
3. What year did you start working in this organization? What year did you start in this particular position?

**REQUEST TO TURN ON RECORDERS AT THIS POINT IN THE INTERVIEW**

| **S.No.** | **Questions** | **Probes** |
| --- | --- | --- |
|  | Comment freely on the prevalence of TB among different age groups and gender in the last 5 years in your LGA | - *If increasing in the last 5 years, probe for reasons why* - *If decreasing in the last 5 years, probe for reasons why* - *If the prevalence has been stable in the last 5 years, probe for reasons why* - *Request for documented evidence (service statistics/records/reports)* |
|  | Can you explain how TB services are provided in your LGA and its influence on client satisfaction and treatment outcomes? | - *Approach for TB service provision* - *Client load* - *Client Satisfaction with quality of care* - *Schedules for community mobilization for early TB screening* - *Overall coordination and frequency of community outreach programmes* - *Effect of Community level interventions/outreach programmes on TB service utilization* - *Mechanism to solicit patient feedback* - *Adequacy and functionality of infrastructural facilities* - *Annual Performance Targets for TB care and service provision* |
|  | Which of the Tuberculosis treatment guidelines and protocols are you aware of?  To what extent have these been utilized by Health workers to guide TB service provision? | - *Availability and use of the following treatment guidelines*   *(Adult TB; Pediatric TB; MDR/XDR; TB/* *HIV co- infection; Infection prevention)*   - *When last were the treatment guidelines updated* - *Update/refresher training for health providers on the use of the guidelines* - *Extent of distribution and use at facilities* - *Potential barriers to provider adherence to the use of the guidelines* - *MDR TB notification systems; infection control policies* |
|  | In your views, does the LGA have adequate skilled staff for TB care and service provision? | - *Adequacy of staff by cadres* - *Frequency of training/refresher training and type of training ( i.e. pediatric TB treatment, adult TB treatment, MDR TB management, management and supervisory skills course, TB managers course, laboratory, infection prevention and control and community activities).* - *Supervision and monitoring systems*. |
|  | Can you explain the extent to which the resources for TB diagnostic and laboratory services are available in your LGA. | - *Availability of trained Laboratory technician on the use of recommended TB diagnostic test* - *Laboratory logistics and supplies* - *Turn-around times for lab results -* *sputum microscopy* |
|  | Please describe the adequacy of the National Health Information System for Tuberculosis care and service provision? | Probe for   - *Availability and actual use of registers* - *Extent of submission of data reporting tools to the next level and frequency of written of oral feedback from national level* - *Frequency of Data audit/assessment at facilities and LGA levels* - *Data quality issues* |
|  | Can you describe the drug and supplies logistics system for your LGA and gaps or strengths with regards to its effective management | Probe for   - *Availability of anti TB drugs* - *Standardized protocol for forecasting TB drugs* - *Mechanisms for forecasting, procurement, and distribution of drugs;* - *Supervisory mechanism for forecasting, procurement, and drug distribution* - *Episodes and duration of stock out in the last 6 months and mitigation efforts* |
|  | What are the challenges hindering TB delivery services? | - *Barriers to quality TB service provision -Probe for case detection and treatment* - *Probe for challenges at health facility and community levels?* |
|  | What would you recommend for improved TB service provision? | Probe:  *Staffing, Drug and Supplies, Health Information System, Infrastructural Facilities, Health financing etc*  *Ways to improve patients’ adherence to treatment, Improve Case detection and DOTS coverage* |

**Thank the interviewee for participation!**

***QUALITY OF TUBERCULOSIS SERVICE DELIVERY AND TREATMENT OUTCOMES IN NIGERIA***

**KEY INFORMANT INTERVIEW FOR TB FOCAL PERSON AT THE FACILITY**

**Introduction**

I am here on behalf of the College of Medicine, University of Ibadan, Nigeria. The goal of this study is to evaluate the quality of TB service delivery and outcomes by cadres of health workers and types of health facilities in the two Nigerian states.Your participation is entirely voluntary and your decision whether or not to participate will involve no penalty or loss of benefits.

Remember:

- There are no right and wrong answers, this is not an examination.
- Please answer all the questions as honestly and accurately as you can — this is very important and help improve the delivery of TB care in this state.

If you have further questions or concerns, please contact the undersigned

**Professor Ademola Ajuwon (Principal Investigator), Department of Health Promotion and Education, Faculty of Public Health, College of Medicine, University of Ibadan, Nigeria. Email:ajajuwon@yahoo.com; Phone number: 08034892561**

**Note to Interviewer: *Share the study information sheet and request for signed consent***

**Demographics, TAKE NOTES** (identifying information to be kept separate from interview transcripts)

Just to confirm that I have your details right…..

1. Participant’s name & organization and email/ contact details (fill in beforehand if possible):
2. Participant’s title/designation and primary responsibilities:
3. What year did you start working in this organization? What year did you start in this particular position?

**REQUEST TO TURN ON RECORDERS AT THIS POINT IN THE INTERVIEW**

| **S.No.** | **Questions** | **Probes** |
| --- | --- | --- |
|  | Comment freely on the prevalence of TB among different age groups and gender in the last 5 years in your facility | - *If increasing in the last 5 years, probe for reasons why* - *If decreasing in the last 5 years, probe for reasons why* - *If the prevalence has been stable in the last 5 years, probe for reasons why* - *Request for documented evidence( service statistics/records/reports)* |
|  | Can you explain how TB services are provided in your facility and its influence on client satisfaction and treatment outcomes? | - *Approach for TB service provision* - *Client load* - *Client Satisfaction with quality of care* - *Schedules for community mobilization for early TB screening* - *Overall coordination and frequency of community outreach programmes* - *Effect of Community level interventions/outreach programmes on TB service utilization* - *Mechanism to solicit patient feedback* - *Adequacy and functionality of infrastructural facilities* - *Annual Performance Targets for TB care and service provision* |
|  | Which of the Tuberculosis treatment guidelines and protocols are you aware of?  To what extent have these been utilized by Health workers to guide TB service provision? | - *Availability and use of the following treatment guidelines*   *(Adult TB; Pediatric TB; MDR/XDR; TB/* *HIV co- infection; Infection prevention)*   - *When last were the treatment guidelines updated* - *Update/refresher training for health providers on the use of the guidelines* - *Extent of distribution and use at facilities* - *Potential barriers to provider adherence to the use of the guidelines* - *MDR TB notification systems; infection control policies* |
|  | In your views, does the facility have adequate skilled staff for TB care and service provision? | - *Adequacy of staff by cadres* - *Frequency of training/refresher training and type of training ( i.e. pediatric TB treatment, adult TB treatment, MDR TB management, management and supervisory skills course, TB managers course, laboratory, infection prevention and control and community activities).* - *Supervision and monitoring systems*. |
|  | Can you explain the extent to which the resources for TB diagnostic and laboratory services are available in your facility. | - *Availability of trained Laboratory technician on the use of recommended TB diagnostic test* - *Laboratory logistics and supplies* - *Turn-around times for lab results -* *sputum microscopy* |
|  | Please describe the adequacy of the National Health Information System for Tuberculosis care and service provision? | Probe for   - *Availability and actual use of registers* - *Extent of submission of data reporting tools to the next level and frequency of written of oral feedback from national level* - *Frequency of Data audit/assessment at facilities* - *Data quality issues* |
|  | Can you describe the drug and supplies logistics system for your facility and gaps or strengths with regards to its effective management | Probe for   - *Availability of anti TB drugs* - *Standardized protocol for forecasting TB drugs* - *Mechanisms for forecasting, procurement, and distribution of drugs;* - *Supervisory mechanism for forecasting, procurement, and drug distribution* - *Episodes and duration of stock out in the last 6 months and mitigation efforts* |
|  | What are the challenges hindering TB delivery services? | - *Barriers to quality TB service provision -Probe for case detection and treatment* - *Probe for challenges at health facility and community levels?* |
|  | What would you recommend for improved TB service provision? | Probe:  *Staffing, Drug and Supplies, Health Information System, Infrastructural Facilities, Health financing etc*  *Ways to improve patients’ compliance to treatment, Improve Case detection and DOTS coverage* |

**Thank the interviewee for participation!**
